# Mechanisms of neuromodulatory volume transmission

**DOI:** 10.1038/s41380-024-02608-3

**Published:** 2024-05-24

**Authors:** Özge D. Özçete, Aditi Banerjee, Pascal S. Kaeser

**Affiliations:** Department of Neurobiology, Harvard Medical School, Boston, MA 02115 USA

**Keywords:** Neuroscience, Molecular biology

## Abstract

A wealth of neuromodulatory transmitters regulate synaptic circuits in the brain. Their mode of signaling, often called volume transmission, differs from classical synaptic transmission in important ways. In synaptic transmission, vesicles rapidly fuse in response to action potentials and release their transmitter content. The transmitters are then sensed by nearby receptors on select target cells with minimal delay. Signal transmission is restricted to synaptic contacts and typically occurs within ~1 ms. Volume transmission doesn’t rely on synaptic contact sites and is the main mode of monoamines and neuropeptides, important neuromodulators in the brain. It is less precise than synaptic transmission, and the underlying molecular mechanisms and spatiotemporal scales are often not well understood. Here, we review literature on mechanisms of volume transmission and raise scientific questions that should be addressed in the years ahead. We define five domains by which volume transmission systems can differ from synaptic transmission and from one another. These domains are (1) innervation patterns and firing properties, (2) transmitter synthesis and loading into different types of vesicles, (3) architecture and distribution of release sites, (4) transmitter diffusion, degradation, and reuptake, and (5) receptor types and their positioning on target cells. We discuss these five domains for dopamine, a well-studied monoamine, and then compare the literature on dopamine with that on norepinephrine and serotonin. We include assessments of neuropeptide signaling and of central acetylcholine transmission. Through this review, we provide a molecular and cellular framework for volume transmission. This mechanistic knowledge is essential to define how neuromodulatory systems control behavior in health and disease and to understand how they are modulated by medical treatments and by drugs of abuse.

Neuromodulatory transmission broadly controls neural circuits and behavior. It is exceptionally diverse and includes many different types of transmitters [1–5]. For example, several monoaminergic systems and many neuropeptides regulate nervous system function. Disruption or dysfunction of modulatory transmission is broadly implicated in brain disease, ranging from neurodevelopmental and neuropsychiatric disorders to neurodegeneration and brain cancer. Furthermore, many medical treatments and drugs of abuse directly act on these systems. Hence, detailed molecular, cellular and systems level understanding of neuromodulation is a central goal of current neuroscience.

The main ascending modulatory systems signal through three monoamines, dopamine, norepinephrine, and serotonin. They also include the central cholinergic system. Neuropeptides, amino acid transmitters, additional monoamines (histamine, epinephrine, and melatonin), endocannabinoids, and other transmitters can also operate through volume transmission [2–4, 6]. Here, we focus on the ascending modulatory systems and assess their release machineries, their transcellular organization, and their transmission scales. We then discuss volume transmission features of amino acid transmitters and neuropeptides. We identify five domains through which these modulatory systems are specified, and assess them in view of the rich, mechanistic knowledge that has been developed for synaptic transmission.

## Synaptic and volume transmission

**Fig. 1:**
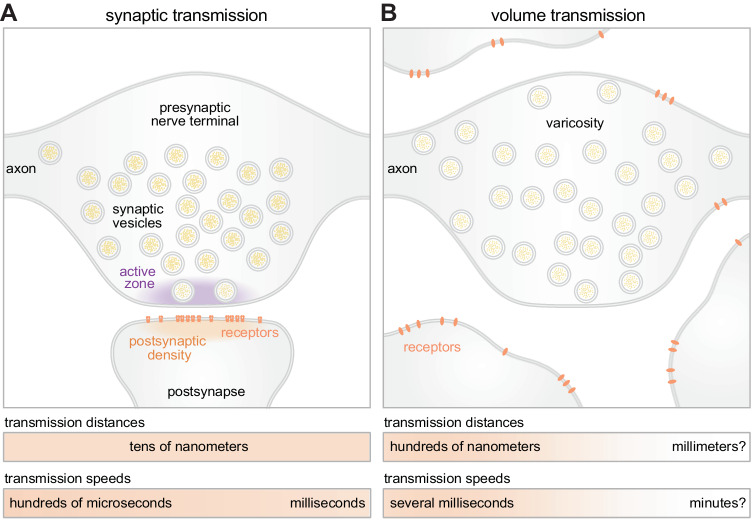
Synaptic and volume transmission. Schematic comparison of key features of the organization of synaptic (**A**) and volume (**B**) transmission. Tight apposition is a hallmark of synapses. Volume transmission lacks this organization and release-receptor distances are often much larger than at synapses. While synaptic transmission is typically mediated by ionotropic receptors, volume transmission usually operates through G protein-coupled receptors (GPCRs). Estimated transmission distances and speeds are indicated at the bottom and discussed in the text.

The principal difference between synaptic and volume transmission is that volume transmission is not “wired”, meaning that it does not rely on synaptic connections (Fig. 1) [3, 7, 8]. A hallmark feature of synaptic transmission is its temporal and spatial precision [9, 10]. At synapses, secretion is mediated by the SNARE proteins synaptobrevin-2/VAMP2 on vesicles, syntaxin-1 and SNAP-25 on the plasma membrane, and by Munc18. Action potentials trigger SNARE-mediated vesicle fusion via opening of voltage-gated Ca^2+^ channels (Ca_V_s). Synaptotagmins sense the local Ca^2+^ increase to induce fusion of transmitter-laden vesicles with the plasma membrane. Synaptic vesicle exocytosis is restricted to a region at the presynaptic plasma membrane called the active zone that contains RIM, RIM-BP, Munc13, ELKS, Liprin-α, and Piccolo/Bassoon [11, 12]. Active zone protein machinery tethers vesicles to their future sites of release, primes them to render the vesicles fusion-ready, orchestrates Ca_V_ positioning, and controls the operation of SNARE proteins and Ca^2+^ sensors. Through this organization, action potentials trigger release without delay, and transmitters are detected postsynaptically by ionotropic receptors that are precisely apposed to presynaptic release sites. Release-receptor distances are short, tens of nanometers, and the synaptic cleft forms a contained space that limits diffusion of transmitter away from the cell-to-cell contact. This synaptic structure warrants that signal transmission occurs rapidly, within ~1 ms of a presynaptic action potential, that a vesicular fusion event reliably leads to postsynaptic receptor activation, and that intercellular signaling is restricted to the two neurons participating in the synapse.

Early electron microscopic studies found that neuromodulatory nerve terminals, often called varicosities, do not form synaptic appositions in most cases [13–18]. In parallel, immunostainings revealed poor colocalization between neuromodulatory axons and their corresponding receptors [19–21]. The asynaptic nature of varicosities and the release-receptor mismatch led to the conclusion that modulatory systems act over distance. These observations defined volume transmission as a signaling mode, yet precise mechanisms have been difficult to establish. This is in part because different modulatory systems employ distinct mechanisms, and transmission distances and speeds vary greatly (Fig. 1). Furthermore, finding an overarching and sharp definition of volume transmission has been complicated by the fact that there isn’t a morphological correlate like the synaptic structure.

In this review, we focus on the discussion of volume transmission as vesicular transmission that is not restricted to a synaptic contact. A single vesicular release event might act on multiple cells as long as their receptors are positioned close enough to be reached by transmitter diffusion. Volume transmission can be defined more broadly to include non-vesicular forms of release and many additional characteristics have been assigned to it [8, 22]. Properties like enhanced transmission delays, low specificity of coded information, and low energy cost have been proposed as defining qualities, but synaptic and volume transmission can share these features. The model of persistent ambient (“tonic”) transmitter levels, often detected with microdialysis, has been a point of focus in volume transmission. However, vesicular release generates a dynamic signal. When a vesicle fuses, transmitter originates from a point source and it is rapidly diluted in the extracellular volume. The transmitter signal is further shaped by buffering, reuptake, and degradation. Hence, “tonic” transmitter levels are highly dynamic in space and time. These dynamics matter for receptor activation as they generate massive spatiotemporal transmitter gradients. The detection of seemingly constant ambient levels arises from low sampling frequency and large collection volumes, and they are composed of highly dynamic signals even at baseline, as we recently discussed for dopamine [23].

**Fig. 2:**
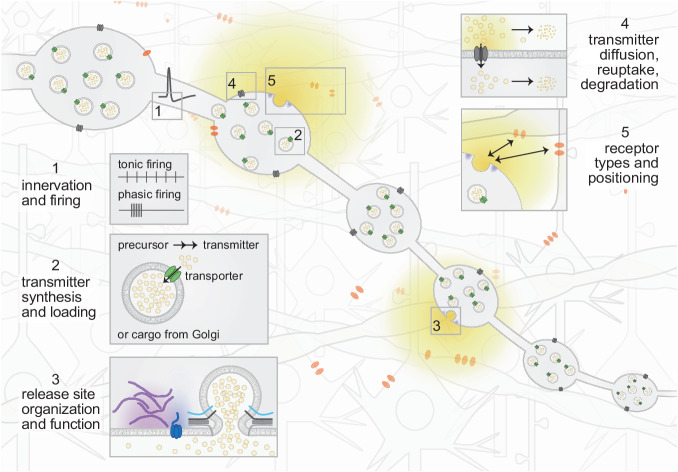
Domains that diversify transmission systems. Schematic display of five domains that differentiate volume transmission systems from synaptic transmission and from one another. The modulatory systems we discuss in the review are evaluated considering these domains.

Fundamentally, volume transmission systems can differ from classical synaptic transmission and from one another in five domains (Fig. 2): (1) the innervation patterns and firing properties of the neurons, (2) the subcellular localization of transmitter synthesis and transmitter loading into different types of vesicles, (3) the architecture of axonal release sites and release site density along an axon, (4) the balance between diffusion, reuptake and degradation of the released transmitter, and (5) the types of transmitter receptors and their relative positioning to transmitter release sites. While we focus on transmission mediated by axonal release, neurons secrete transmitter from somata and dendrites as well [24, 25]. We complement the review with important insights from the characterization of somatodendritic transmission.

We evaluate the prototypical neuromodulator dopamine considering these domains and examine how norepinephrine and serotonin might be similar or differ. We then discuss neuromodulators that also act as synaptic transmitters and neuropeptides. We focus on the transmission mode between cells without evaluating signaling pathways in target cells. While we emphasize neuron-to-neuron transmission, the principles we discuss can involve non-neuronal cells as targets or as regulators of signaling. Finally, neuromodulatory neurons often release multiple transmitters; we refer to previous reviews on the topic of co-transmission [26, 27].

## Key lessons from dopamine

### Dense striatal innervation and switches in firing modes of midbrain dopamine neurons

Most brain dopamine is synthesized by neurons in the substantia nigra and ventral tegmental area that send their projections to the striatum, prefrontal cortex, amygdala, hippocampus and other brain areas [23, 28, 29]. Their axonal arbors are extensively branched. In the striatum, massive arborization leads to dense innervation with an estimated ~3% of the striatal volume occupied by dopamine axons [30]. In addition to axonal release, dopamine cell bodies and dendrites also release dopamine through somatodendritic exocytosis [24].

Dopamine neurons exhibit three modes of action potential firing [31, 32]. Tonic (or pacemaker) firing is cell-autonomous at frequencies of 0.2–10 Hz [33]. Phasic (or burst) firing entails 3 to 10 action potentials at >10 Hz, and shared somatodendritic inputs synchronize this mode across dopamine neurons [34, 35]. Action potentials can also be induced in distal dopamine axons by cholinergic interneurons via the activation of nicotinic acetylcholine receptors (nAChRs) on dopamine axons [32, 36].

Changes in firing rates regulate striatal dopamine dynamics and behavior [23, 31, 37–40]. One recent model, the domain-overlap model [23], proposed that baseline activity leads to isolated and local vesicular release events with activation of nearby receptors. Shared inputs induce burst firing in groups of dopamine neurons, and this leads to activation of receptors that are only reached through overlapping dopamine domains from multiple release sites. The third mechanism, firing induced in distal axons by cholinergic interneurons, was only recently discovered [32]. It remains uncertain how it contributes to striatal regulation in vivo [41, 42], how it intersects with ascending action potentials, and how it influences somatic dopamine neuron activities. It might selectively modulate striatal dopamine or regulate somata and dendrites in the midbrain through backpropagation [32, 43]*.*

### Synthesis and vesicular loading of dopamine

**Fig. 3:**
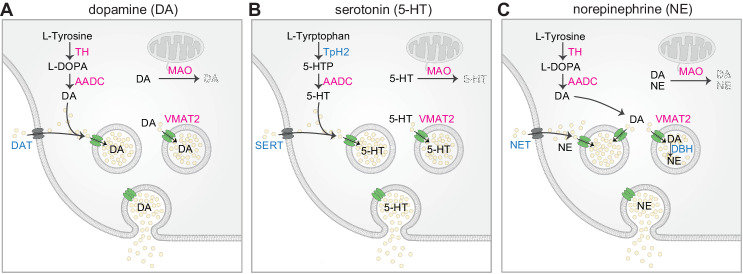
Monoamine synthesis, loading, reuptake, and degradation. Schematic of the metabolism of dopamine (**A**), serotonin (**B**) and norepinephrine (**C**) with shared (pink) and distinct (blue) steps highlighted. Monoamines are synthesized from amino acids through enzymatic reactions. The vesicular transporter VMAT2 is shared across the three central monoamines, while reuptake is mediated by a specific transporter for each monoamine. Intracellular degradation is mediated by MAO for all three discussed monoamines; 5-HT (5-hydroxytryptamine, serotonin), 5-HTP (5-hydroxytryptophan), AADC (aromatic l-amino acid decarboxylase), DA (dopamine), DAT (dopamine transporter), DBH (dopamine-β-hydroxylase), l-DOPA (l-dihydroxyphenylalanine, levodopa), MAO (monoamine oxidase), NE (norepinephrine), NET (norepinephrine transporter), SERT (serotonin transporter), TH (tyrosine hydroxylase); TpH2 (tryptophan hydroxylase 2), VMAT2 (vesicular monoamine transporter 2).

Dopamine synthesis is cytosolic and occurs in two steps that are in part shared with other monoamines (Fig. 3). The enzyme tyrosine hydroxylase (TH), often used as a marker, converts the amino acid l-tyrosine to l-dihydroxyphenylalanine (l-DOPA), a precursor for dopamine and norepinephrine. Aromatic l-amino acid decarboxylase (AADC) then catalyzes the generation of dopamine from l-DOPA, and dopamine is loaded into vesicles or degraded via monoamine oxidase (MAO) [43–47] (Fig. 3A).

In the brain, vesicular loading of dopamine is mediated by the vesicular monoamine transporter 2 (VMAT2) [48, 49]. Neurons load neurotransmitters into multiple types of vesicles [50]. Synaptic vesicles are ~50 nm in diameter, have a clear core, and are filled locally with neurotransmitter. Large dense core vesicles (LDCVs), as their name infers, are larger (typically 80–120 nm diameter), have a protein-dense core, and are supplied through long-range transport. Dopamine can in principle be loaded into both types of vesicles. In the adrenal gland, dopamine and other catecholamines are stored in LDCVs and released from these vesicles upon stimulation [51–53]. In striatal dopamine axons, dopamine is mostly loaded into small, clear vesicles [54, 55]. First, the axon is filled with clear vesicles and LDCVs are overall sparse [17, 56–58], although some varicosities contain clear vesicles that are larger than synaptic vesicles [56]. Second, in dopamine neurons, VMAT2 is preferentially present on small, clear vesicles [59, 60]. Third, dopamine release is strongly dependent on proteins that are typical for synaptic vesicles, for example synaptotagmin-1 (Syt-1) and synaptobrevin-2/VAMP2 [61–64]. Finally, as discussed in the next section, the release properties of dopamine are similar to those of classical neurotransmitters stored in synaptic vesicles. Hence, most striatal dopamine is released from synaptic vesicles.

The intracellular compartment for somatodendritic dopamine release remains unclear. Early work suggested release from tubulovesicular structures rather than vesicles [65–67]. A recent study has found that the vesicular Ca^2+^ sensor Syt-1 is important for somatodendritic exocytosis [68], perhaps suggesting the involvement of synaptic vesicles.

### Sparse dopamine release sites confer a high vesicular release probability

Action potentials trigger vesicular dopamine release, which relies on SNAREs [61, 62] and on extracellular Ca^2+^ [64, 69, 70]. At classical synapses, Ca^2+^ triggering is fully mediated by Ca_V_2 channels with major roles for Ca_V_2.1 (P/Q-type) and Ca_V_2.2 (N-type), and contributions of Ca_V_2.3 (R-type) are limited [9, 10]. In contrast, the Ca_V_2-dependence of dopamine release is partial, and Ca_V_1 (L-type) and Ca_V_3 (T-type) channels also contribute [32, 71]. Dopamine release occurs with synchrony to increase local dopamine concentration within milliseconds of axonal depolarization, and dopamine secretion has a high vesicular release probability [63, 72]. This suggests the presence of molecular machinery to organize these processes.

**Fig. 4:**
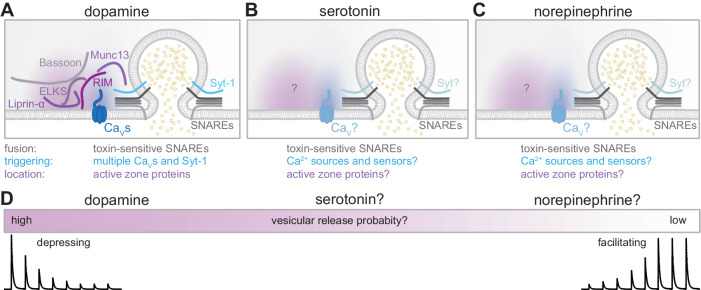
Mechanisms and properties of monoamine release. Schematics of the proteins that mediate the release of dopamine (**A**), serotonin (**B**) and norepinephrine (**C**). Evidence for the importance of active zone proteins, Ca^2+^ sources and Ca^2+^ sensors stems from conditional mouse gene knockouts; evidence for the importance of SNARE proteins stems from cleavage with bacterial neurotoxins; evidence for the importance of voltage-gated Ca^2+^ channels (Ca_V_s) is further supported by pharmacological blockade. Properties (**D**) of the release of dopamine, serotonin, and norepinephrine as assessed in brain slices and in vivo by a variety of techniques. These monoamines show a range of properties with depression detected in brain slices for dopamine, and facilitation for norepinephrine in some cases.

Recent work indeed established that active zone-like protein architecture is essential for evoked dopamine release (Fig. 4). The scaffold RIM is present in small clusters in dopamine axons, and its ablation from dopamine neurons disrupts evoked release [63]. Moreover, these axons contain Bassoon, ELKS2 and Munc13-1 clustered in release site-like structures, and Liprin-α also contributes to evoked dopamine secretion [63, 73–75]. At synapses, RIM primes vesicles through recruitment and activation of Munc13 [76, 77], it links primed vesicles to Ca^2+^ channels [78, 79], and it couples these processes to the membrane phospholipid PIP_2_ [80, 81]. In dopamine axons, RIM mediates fusion through Munc13 as well [75, 82]. RIM C2B domains, which bind to PIP_2_ and Liprin-α, also participate in dopamine release [75, 80, 83]. Consistent with only partial reliance on Ca_V_2s, and with the dispensability of RIM-BP and ELKS for dopamine release, the RIM sequences that bind to these proteins are not essential for dopamine release [32, 63, 71, 75].

Altogether, these analyses led to the model of active zone-like release sites for rapid, synchronous dopamine secretion with a high vesicular release probability (Fig. 4). Proximity-based proteomic approaches identified additional release site proteins in dopamine axons [84, 85]. One remarkable morphological feature of dopamine axons is that active zone protein clusters are sparse, and ~75% of vesicle-containing varicosities do not have detectable levels of active zone proteins [63, 75]. This matches with functional experiments in which only a subset of dopamine varicosities appears to release dopamine upon stimulation [86].

The synchronous nature of evoked dopamine release indicates the need for Ca^2+^ sensors that rapidly respond to Ca^2+^ entry. At conventional synapses, synchronous release is mediated by Syt-1, -2, or -9, Ca^2+^ sensors with a low Ca^2+^ affinity and fast kinetics [10, 87]. In dopamine neurons, axonal and somatodendritic dopamine release evoked by action potentials depend on Syt-1 [64, 68]. When Syt-1 is ablated, a form of dopamine release that can be induced with strong depolarizations or with action potential trains persists and is mediated by Ca^2+^ sensors that have not been identified yet. Early work revealed a different extracellular Ca^2+^ dependence and suggested reliance on distinct Ca^2+^ sensors for somatodendritic release [69, 88, 89], but it is mediated by RIM and Syt-1 similar to axonal release [68, 90].

### Diffusion and reuptake are key determinants of dopamine transmission

After dopamine is released, local dopamine concentrations are shaped by diffusion and reuptake. Diffusion rapidly dilutes dopamine in the extracellular volume and the dopamine transporter (DAT) mediates reuptake into dopamine neurons [23, 91]. It has remained challenging to measure local dopamine concentrations and dynamics, which chiefly determine dopamine receptor activation. A single dopamine vesicle contains a few thousands to tens of thousands of dopamine molecules, and dopamine concentration inside a vesicle might be as high as ~1 M [91–94]. In consequence, the dopamine concentration at the mouth of a fusing vesicle must be hundreds of millimolar. Dopamine concentration from this point source likely drops to a nanomolar range within a few milliseconds and within micrometers of a fusing vesicle [23, 91, 95]. In most cases, dopamine diffusion is not limited by the constraints of a synaptic cleft, but tortuosity in the extracellular space may limit or direct diffusion [17, 56, 91]. While diffusion dominates within a few micrometers of the fusion event, DAT further shapes the signal through reuptake at longer distances [91, 96, 97]. Currently available detection methods, including electrochemistry, microdialysis and fluorescent sensors, do not allow for measuring these dopamine dynamics at the relevant spatiotemporal nanoscales. Instead, they often average over extended space and/or time.

### Models of dopamine release-receptor appositions

Dopamine is sensed by G protein-coupled receptors (GPCRs) classified into D1-like (D1 and D5; mostly Gα_s_) and D2-like (D2, D3 and D4; mostly Gα_i_) receptors [98]. In the striatum, D1 and D2 receptors together account for most striatal dopamine receptors [95, 99–101]. They are on medium spiny neurons (MSNs) and their cell-type specific expression defines D1- and D2-MSNs, which form the direct (D1-MSNs) and indirect (D2-MSNs) pathways [28, 102]. D1 receptors are also expressed on glial cells and D2 receptors on acetylcholine and dopamine axons (reviewed in [23]).

The relative positioning of release sites and receptors is a critical determinant of receptor activation. Because experimental resources including appropriate microscopy and antibodies to define dopamine release hotspots and dopamine receptor distributions are not readily available, the spatial organization of release and receptors has not been conclusively determined. Hence, models of dopamine transmission have been inferred from properties other than measured release-receptor distances. One important finding is that dopamine release sites are sparse, with only every ~4th varicosity containing active zone-like machinery and with frequent occurrence of varicosities with a limited ability to release [63, 73, 75, 86]. One possibility is that the varicosities which form synaptic appositions are the ones that release dopamine in response to stimulation. Initial electron microscopic studies have found that roughly one quarter of varicosities have synaptic appositions [17], similar to the sparsity of release sites [63, 86]. At these varicosities, dopamine receptors are not found in the apposed postsynaptic densities, but ~100 nm lateral, suggesting that the receptors are not as precisely aligned as ionotropic receptors at synapses [9, 57]. A recent study, however, established that synaptic appositions in dopamine axons are much sparser: only ~1% of the dopamine varicosities have them [56]. This finding makes it highly unlikely that these are the relevant signaling units and support the model that dopamine signaling does not rely on synaptic organization.

Dopamine receptor affinities have been used to develop models on receptor activation and release-receptor organization. Initial studies found that D1 receptors have a lower dopamine affinity (K_d_ ~1 μM) than D2 receptors (K_d_ ~25 nM) [100]. These differences formed the basis for models in which dopamine activates D1 receptors during burst firing close to release sites and D2 receptors sense steady-state tonic dopamine further away. These affinity-based models need to be updated [23, 95, 103, 104]. First, tonic dopamine release is vesicular and generates small, transient “sparks” that have a high dopamine concentration; the “stable” tonic dopamine level of ~2 nM is likely an artifact of sampling that averages over a large space and long times [23]. Second, the affinity measurements may not reflect in vivo receptor affinities, and both types of receptors may have a low affinity [72, 105–107]. Third, receptor positioning cannot be inferred from available data; it has to be measured in relationship to dopamine sources.

Studies of the properties of D2 receptor-induced inhibitory postsynaptic currents (D2-IPSCs) support that D2 receptors are rapidly activated by high, local dopamine concentrations. D2-IPSCs are mediated via coupling of D2 receptors to endogenous (midbrain) or exogenously expressed (striatum) G protein-gated inwardly rectifying potassium (GIRK) channels [72, 108]. In brain slices of both brain areas, D2-IPSCs are readily elicited by single stimuli. D2 receptor activation necessitates ~100 μM dopamine within a short time window after stimulation and D2-IPSCs have fast kinetics with a response onset of tens of milliseconds that is limited by GPCR signaling speeds [72, 108–110]. These studies establish that dopamine needs to rise quickly to a high concentration for D2 receptor activation [23].

In conclusion, the working model is that dopamine is secreted effectively via vesicular exocytosis at sparse sites with major contributions of RIM, Munc13, and Syt-1. Diffusion chiefly determines local dopamine levels and reuptake shapes the signal. Receptor activation strongly depends on the relative positioning of dopamine receptors to release sites, but these spatial relationships remain unknown.

## Are dopamine concepts applicable to central serotonin and norepinephrine transmission?

### Innervation patterns and firing properties

Innervation and firing patterns of norepinephrine and serotonin neurons have similarities with the dopamine system. Most norepinephrine and serotonin neuron somata are in the locus coeruleus and the raphe nuclei, respectively, and their axons project throughout the brain [111–114]. Compared with the exceptionally high striatal dopamine innervation, norepinephrine and serotonin axons are less dense in their target areas [114, 115]. Similar to dopamine, norepinephrine and serotonin are released from axonal varicosities and synaptic appositions are largely absent [13–15, 17, 116–118].

Firing properties are shared across monoaminergic systems. Serotonin neurons fire tonically at 0.03–3 Hz and in bursts up to 17 Hz, and these firing modes can encode distinct information [119–122]. Norepinephrine neurons also switch between tonic and phasic firing to modulate behavioral states [123–125]. Repetitive stimulation of serotonin and norepinephrine neurons increases extracellular transmitter levels [126–128], and how firing shapes release and signaling is discussed further below.

### Synthesis and vesicular loading

Most norepinephrine and serotonin are released from vesicles in an action potential- and Ca^2+^-dependent manner. This is supported by the sensitivity of release to Ca^2+^ removal, to toxins that cleave SNARE proteins, and to blockade of vesicular loading [127, 129–137]. VMAT2 is the shared vesicular transporter for dopamine, norepinephrine, and serotonin (Fig. 3). Constitutive VMAT2 ablation in mice, like that of the synthesis enzymes TH and dopamine β-hydroxylase (DBH), is lethal [138–140], while conditional VMAT2 knockout in serotonin or norepinephrine neurons depletes the corresponding transmitters but does not induce lethality [141–143].

Norepinephrine is synthesized from l-tyrosine via dopamine (Fig. 3C). After the first two enzymatic steps with TH and AADC, dopamine is loaded into vesicles by VMAT2. The intravesicular enzyme DBH then converts dopamine to norepinephrine and DBH is partially co-released with the transmitter [144, 145]. Because norepinephrine is synthesized from dopamine, the commonly used marker TH labels both types of neurons. Serotonin is synthesized in the cytosol from its precursor l-tryptophan (Fig. 3B) via two steps involving the brain-specific tryptophan hydroxylase 2 (TpH2), and AADC [146].

It remains uncertain which vesicle types mediate norepinephrine and serotonin release in the vertebrate CNS. Retzius cells of the leech are mechanosensory neurons that use serotonin as the main transmitter. There, serotonin is released from small, clear vesicles and from LDCVs [147, 148]. These vesicles are estimated to discharge ~5000 or ~80,000 serotonin molecules, respectively. Rat brain serotonin and norepinephrine neurons also contain both vesicle types [13], and pharmacological studies supported the presence of multiple compartments for serotonin storage and release [149]. VMAT2 is present on both synaptic vesicles and LDCVs, and its levels may be high on LDCVs of serotonin axons while being mostly on synaptic vesicles in dopamine axons [59, 60, 150]. Together, these studies suggest that multiple vesicle types contribute to norepinephrine and serotonin release, and signaling with different kinetics and functions might be a result of this organization [151].

### Release machinery and release site distribution

While norepinephrine and serotonin release are vesicular, the machineries for their secretion (Fig. 4B, C) and the distribution of release sites within an axon are largely unknown. Botulinum neurotoxin A and tetanus neurotoxin, which cleave SNAREs, inhibit the release of ^3^[H]-labeled norepinephrine and serotonin from synaptosomes or in brain slices [130, 131, 134]. Despite the similar SNARE-dependence of norepinephrine, serotonin and dopamine release, studies using electrochemistry, fluorescent sensors, and mouse genetics suggest differences (Fig. 4D). When imaged with GPCR-based sensors, dopamine and serotonin only moderately build-up during stimulus trains [152, 153], consistent with the depression observed for dopamine with amperometry or D2-IPSCs in brain slices [63, 72] (Fig. 4D). While dopamine release in response to stimulus trains is enhanced upon knockout of the vesicle-associated protein Synapsin, serotonin release appears to be unaffected [154]. In hippocampal interneurons, serotonin elicits ionotropic, 5HT_3_-receptor-mediated responses within a few milliseconds after a single stimulus [155]. Norepinephrine release often necessitates stimulus trains for effective detection with voltammetry or fluorescent sensors, suggesting that release might facilitate and might have slower kinetics (Fig. 4D) [156–158]. In contrast, whole-cell recordings effectively detect single stimulus-evoked IPSCs mediated by somatodendritic release that activates adrenergic receptors [109]. Overall, these studies indicate that the release machineries for these monoamines might differ between transmitters and cellular compartments.

The strong extracellular Ca^2+^ dependence of evoked serotonin and norepinephrine release suggests that voltage-gated Ca^2+^ channels mediate their release [127, 129, 135, 137, 159]. Indeed, blockers of Ca_V_2.1 and Ca_V_2.2 impair norepinephrine and serotonin release detected by high-performance liquid chromatography, microdialysis, or as ^3^[H]-labeled transmitter [160–164]. Because of the low spatiotemporal resolution of these methods, they do not establish that Ca^2+^ entry through these channels directly triggers vesicular exocytosis. Different from dopamine release, both norepinephrine and serotonin release appear to be resistant to Ca_V_1 blockade [137, 159, 165]. Finally, conditional ablation of Ca_V_2.1 in serotonin neurons increased the firing rate of these neurons and impacted mouse behavior, but it was not assessed whether Ca_V_2.1 is involved in serotonin release [166].

Optical tracer experiments suggested that norepinephrine axons have many varicosities that are not release-competent, and morphological studies revealed that the density of varicosities in norepinephrine axons differs across brain areas [167–169]. Hence, heterogeneity in axonal organization might contribute to variable signaling properties within an axon and across brain areas.

Future studies should assess the composition and distribution of release machinery in serotonin and norepinephrine axons and determine the resulting release and signaling properties. Recent advances in the development of fluorescent sensors for detecting serotonin and norepinephrine provide tools for answering these questions [153, 156, 170–173].

### Diffusion and reuptake

Once released, the balance between diffusion and reuptake determines neurotransmitter concentration at the corresponding receptors. Extracellular serotonin levels measured by voltammetry reach micromolar concentrations upon stimulation [127]. The local serotonin concentration is likely much higher for a brief amount of time in the vicinity of a fusing vesicle given the high intravesicular serotonin concentration [147, 148]. Norepinephrine transporter (NET) and serotonin transporter (SERT) move the corresponding transmitters back into cells. Transporter expression is often limited to the neurons that release the specific transmitter [174, 175], and cross-talk between transmitters and transporters can occur [176]. After reuptake, monoamines are either loaded back into vesicles via VMAT2 or degraded by MAO (Fig. 3) [44–46]. For some neuromodulators, degradation in the extracellular space controls transmitter concentration [177–179]. In contrast, monoamine degradation is intracellular and does not directly contribute to clearing the extracellular space. Monoamine reuptake and degradation mechanisms are targeted by tricyclic antidepressants (TCAs), by selective serotonin reuptake inhibitors (SSRIs), by cocaine (which inhibits DAT, SERT, and NET), and by MAO inhibitors [175, 180, 181].

For striatal dopamine, modeling of the competition between diffusion and reuptake indicates that DAT shapes signaling by limiting dopamine spread [55, 91]. A key factor is the high density of DAT-containing dopamine axons in the striatum. Because serotonin and norepinephrine axons are much less dense, the corresponding transporters do not cover a large fraction of the extracellular volume and the transmitters are less likely to encounter transporters as they diffuse away from the release site. Hence, reuptake might be less powerful in shaping the signaling in sparse modulatory systems [127, 136, 158].

### Receptors and release-receptor appositions

Serotonin and norepinephrine exert diverse functions via large receptor families expressed in brain-area and cell-type specific manners. Most receptors are GPCRs that are widely expressed on neurons and glial cells, and they can help excite or inhibit their targets [182–187]. One exception is the ionotropic 5HT_3_ receptor, through which serotonin acts as a fast excitatory transmitter [155, 188, 189].

Serotonin receptors are organized into seven classes (5HT_1–7_) with 14 subtypes based on ligand affinities, sequence homology, and intracellular transduction mechanisms: 5HT_1_ (mostly Gα_i_; subtypes 5HT_1A,_ 5HT_1B,_ 5HT_1D,_ 5HT_1E,_ 5HT_1F_), 5HT_2_ (Gα_q_; subtypes 5HT_2A_, 5HT_2B_, 5HT_2C_), 5HT_3_ (ionotropic), 5HT_4_ (Gα_s_ and Gα_i_), 5HT_5_ (Gα_i_; subtypes 5HT_5A_, 5HT_5B_), 5HT_6_ (Gα_s_), and 5HT_7_ (Gα_s_) [186, 187]. In many brain areas, the density of serotonin innervation corresponds to receptor density, but mismatches exist [19]. Receptor autoradiography, immunostainings, and electron microscopy found associations between serotonin axons and 5HT_2A_ receptors, but synaptic appositions are overall rare [13, 15, 20, 21, 190]. In the parietal cortex, for example, the mean varicosity to receptor distance is estimated to be ~8 μm, and in the hippocampus, dense receptor staining is present despite sparse serotonin axonal innervation [21].

Two classes of norepinephrine receptors, α- and β-adrenergic receptors, are divided into 9 subtypes: α1 (Gα_q;_ subtypes α_1A_, α_1B_ and α_1D_), α_2_ (Gα_i_; subtypes α_2A_, α_2B_ and α_2C_), and β_1_ to β_3_ (Gα_s_ and Gα_i_) [185, 191]. Norepinephrine receptors show non-uniform expression, and there are mismatches with broad receptor expression but low norepinephrine fiber density, or vice versa [19, 183, 184, 192, 193].

Overall, synaptic appositions are sparse and discrepancy between axon densities and receptor patterns exist [19]. Because the distribution of release sites is uncertain, exact release-receptor associations remain unknown. For striatal dopamine, we proposed the domain-overlap model in which multiple release sites contribute to receptor activation during phasic firing [23]. A similar model might not operate for neuromodulators with a low innervation density because overlap of release domains is less likely to occur. Precise assessment of release site distribution and of the relative positioning of receptors will be essential for understanding functional properties of serotonin and norepinephrine signaling.

## From fast transmitters to neuropeptides: expanding scales of volume transmission

### Fast transmitters

Amino acids are the main synaptic transmitters, but they also act as volume transmitters. For example, extrasynaptic GABA and glutamate receptors detect spillover transmitter from synapses [194–196]. In this case, innervation, vesicle types, and release mechanisms are defined by the synaptic properties, but neurotransmitters escape the synaptic cleft to activate distant receptors for volume transmission. Spillover can operate on ionotropic receptors or on GPCRs. The transmitter can also enter neighboring synapses and activate distant synaptic receptors [197]. It is noteworthy that volume transmission of synaptic neurotransmitters is amplified in states with enhanced activity, for example, during epilepsy. Amino acid transmitters can also be co-released from neurons that use a neuromodulator as their primary transmitter. For example, GABA is co-released with dopamine in the retina and in the striatum [198–200], and this GABA co-transmission shares many properties of dopamine. These examples illustrate that volume transmission is a broad concept, and important forms of signaling can arise as a secondary process from synaptic transmission.

Acetylcholine acts as a synaptic transmitter at the neuromuscular junction. In contrast, acetylcholine is considered a volume transmitter in the central nervous system, although the mode of action remains debated [14, 22, 32, 36, 177, 201–203]. Cholinergic neurons are found in multiple brain regions [204, 205]. Neurons located in the pedunculo-pontine and lateral dorsal tegmental nuclei and in the basal forebrain send ascending projections throughout the brain. In the striatum, local cholinergic interneurons form a dense network, and they account for ~2% of striatal neurons [206, 207]. Their high density, their firing properties and recent work on these interneurons render them well-suited for a discussion of central acetylcholine transmission mechanisms.

**Fig. 5:**
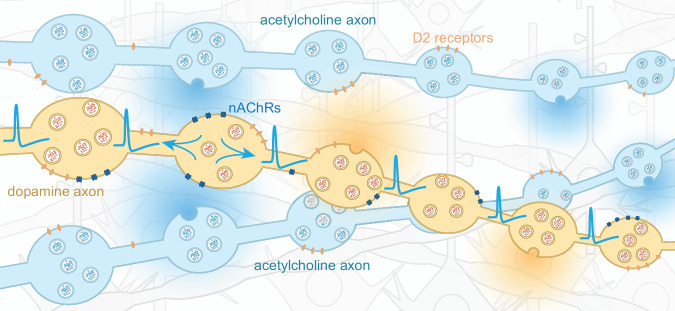
Acetylcholine axon to dopamine axon transmission in striatum. Model of axo-axonal volume transmission through which acetylcholine triggers action potential firing in distal dopamine axons. Acetylcholine is released from local cholinergic interneurons and activates nicotinic acetylcholine receptors (nAChRs) on dopamine axons. This leads to local initiation of firing in dopamine axons. The delay from the time point of cholinergic axon stimulation to detection of dopamine release triggered by an axonal action potential is ~10 ms. Inhibitory dopamine feedback is provided to acetylcholine and dopamine axons via D2 receptors.

In the striatum, cholinergic interneurons activate metabotropic muscarinic acetylcholine receptors expressed on MSNs and on local interneurons. In addition, β2-containing nAChRs are present at high levels on dopamine axons [208]. These ionotropic nAChRs allow for a rapid readout of released acetylcholine and robustly trigger dopamine release [32, 36, 63, 209–213]. The axons of the cholinergic interneurons spread over a large striatal area [214], and the neurons have tonic firing activity, pause-rebound responses, and phasic firing similar to monoamine neurons [215–217]. Ultrastructural characterization suggested that synaptic contacts of striatal cholinergic varicosities are very sparse if existent at all [16, 18], and recent superresolution microscopic studies found that the contact frequency between cholinergic and dopaminergic axons is not higher than a predicted random association given their local densities [32]. Despite this sparse contact frequency, stimulation of cholinergic interneurons powerfully and rapidly, within a few milliseconds, depolarizes the dopamine axonal plasma membrane to induce axonal action potential firing (Fig. 5) [32]. Furthermore, individual cholinergic events that are resistant to action potential blockade can be recorded from dopamine axons as subthreshold depolarizations, and even spontaneous events can lead to dopamine axon action potential firing [36]. Released acetylcholine is rapidly degraded by acetylcholine esterase which is present at very high levels in the striatum [218]. Inhibiting acetylcholine esterase slows the decay kinetics of spontaneous events and leads to a transient increase in event frequency recorded from dopamine axons [36], suggesting that degradation limits acetylcholine spread.

Further insight on acetylcholine transmission comes from electrophysiological recordings of D1-MSNs after expression of GIRK channels. This leads to M4 muscarinic receptor-coupled IPSCs (M4-IPSCs) [203], similar to D2-IPSCs [72, 108], and provides an electrophysiological readout of acetylcholine transmission. Despite the low synaptic connectivity [16], spontaneous unitary M4-IPSCs occur, and M4-IPSCs are sensitive to action potential blockade. M4-IPSCs have a lag of ~40 ms and a rise time of ~80 ms, consistent with the time course of GPCR signaling, and they are rapidly depressing during repetitive stimulation. Acetylcholine esterase inhibition slows M4-IPSC kinetics, suggesting that receptor activation upon diffusion is limited by acetylcholine degradation. While M4 receptors can be positioned on MSN spines [219], it is unclear whether M4 receptor clusters are apposed to acetylcholine releasing nerve terminals. These findings argue against the importance of tonic ambient levels of acetylcholine, but point toward rapid, metabotropic transmission.

In summary, current data indicate that central cholinergic signaling is likely asynaptic and mediated by volume transmission. Like dopamine [23, 72, 108], it has functional features of fast transmission including the induction of spontaneous events and rapid action in the target cell. Striatal acetylcholine secretion might therefore rely on protein machinery that supports high release synchrony and probability. The frequency and architecture of release sites, and their organization relative to various types of acetylcholine receptors remain major open questions. Altogether, work on striatal acetylcholine suggests that volume transmission may modulate target cells rapidly.

### Neuropeptides

Neuropeptide signaling is widespread and diverse with more than 100 neuropeptides, and it regulates behavior with broad spatiotemporal characteristics [4, 5, 220]. These modulators are typically released from LDCVs, and their transmission is mediated by GPCRs. A small number of neurons located in the hypothalamus use neuropeptides as their primary transmitter, while most central neurons release one or multiple neuropeptides from LDCVs in addition to their synaptic transmitter. Hence, for most neuropeptide signaling, innervation patterns and activities reflect the broad range of properties of the neurons that release fast transmitters and co-release neuropeptides.

LDCVs are heterogeneous and are located in somata, dendrites and axons. In contrast to the local production of monoamines and amino acid transmitters, neuropeptides are produced in the soma and supplied via long-range transport [50, 221]. Most synapses contain a very low number of LDCVs, making it challenging to study LDCV exocytosis. Their release generally necessitates strong stimulation, depends on Ca^2+^ and voltage-gated Ca^2+^ channels, and occurs at synapses and extrasynaptically [222–226].

In vertebrate neurons, neuropeptide release machinery has been studied with loss-of-function approaches combined with various release measurements in cultured cells and in brain slices (Table 1). The fundamental message of this body of work is that proteins for synaptic vesicle release also mediate fusion of neuropeptide-containing LDCVs, although the exact properties differ qualitatively and quantitatively. One specific finding is that, different from synaptic vesicle exocytosis, Rab3 is essential for neuropeptide release and operates via its effector RIM [227]. Interestingly, distinct secretory pathways for multiple neuropeptides can exist within a neuron. In olfactory mitral cells, IGF-1 release is mediated by Syt-10, while release of other LDCVs and synaptic vesicles depends on Syt-1 [228, 229]. This heterogeneity of release pathways within a neuron might amplify the signaling capacity greatly.

**Table 1:** Proteins involved in neuropeptide release.

Proteins	Protein family	Neuropeptide, cell type and/or release measurement	Effects	References
CAPS1 and 2	Release site proteins and other regulators	Neurotrophin-3 release assessed with enzyme-linked immunoassay (ELISA) in cultured cerebellar neurons; BDNF-pHluorin, Semaphorin-3A-pHluorin, NPY-pHluorin, or NPY-mCherry release in cultured hippocampal neurons	Knockout of CAPS1 and/or CAPS2 impairs release to various degrees	[239–242]
Complexin1 and 2	Release site proteins and other regulators	IGF-1 levels assessed with ELISA, Syt-10-pHluorin in cultured olfactory bulb neurons; BDNF-quantum dot and BDNF-EGFP release in cultured hippocampal neurons	Knockdown impairs release	[229, 243]
Dynamin 1, 2, and 3	Release site proteins and other regulators	NPY-pHluorin and BDNF-pHluorin in cultured hippocampal neurons	Knockout impairs release	[244]
Munc13-1 and -2	Release site proteins and other regulators	Semaphorin-3A-pHluorin and NPY-pHluorin in cultured hippocampal neurons	Knockout impairs release	[222]
Munc18-1	Release site proteins and other regulators	NPY-pHluorin, BDNF-pHluorin and NPY-mCherry release in cultured hippocampal neurons	Knockout strongly impairs release	[245]
RAB3A, B, C, and D	Release site proteins and other regulators	NPY-pHluorin, BDNF-pHluorin and BDNF release monitored by ELISA in cultured hippocampal neurons	Knockout strongly impairs release	[227]
RIM1 and 2	Release site proteins and other regulators	NPY-pHluorin, BDNF-pHluorin and NPY-mCherry release in cultured hippocampal neurons	Knockout strongly impairs release	[227]
SNAP-25	SNARE proteins	BDNF-pHluorin in cultured cortical neurons; NPY-pHluorin in cultured hippocampal neurons	Knockdown or knockout strongly impair release	[246, 247]
SNAP-47	SNARE proteins	BDNF-pHluorin in cultured cortical neurons	Knockdown impairs release	[246], but see [247]
Synaptobrevin-2/Vamp2	SNARE proteins	BDNF-pHluorin in cultured cortical neurons	Knockdown strongly impairs release	[246]
Synaptotagmin-1	Synaptotagmins	NPY-pHluorin in cultured hippocampal neurons	Knockdown or knockout impairs release	[248]
Synaptotagmin-4	Synaptotagmins	BDNF levels assessed with ELISA in cultured hippocampal neurons	Knockout mildly increases release	[223]
Synaptotagmin-6	Synaptotagmins	BDNF-quantum dot and BDNF-EGFP release in cultured hippocampal neurons	Knockdown impairs BDNF-quantum dot release but not BDNF-EGFP release	[243]
Synaptotagmin-7	Synaptotagmins	NPY-pHluorin in cultured hippocampal neurons	Knockout impairs release	[248]
Synaptotagmin-9	Synaptotagmins	Substance P-pHluorin in cultured striatal neurons	Knockout impairs release	[249]
Synaptotagmin-10	Synaptotagmins	IGF-1 levels assessed with ELISA in cultured olfactory bulb neurons	Knockout impairs release	[228]
Vti1a and b	SNARE proteins	NPY-pHluorin in cultured hippocampal neurons	Knockout strongly impairs release; effects are due to Golgi sorting function	[250]

The table focuses on loss-of-function experiments combined with neuropeptide release measurements in central vertebrate neurons. Proteins are subdivided into SNARE proteins, synaptotagmins (Ca^2+^-sensing and other isoforms), and release site proteins including other regulators.

Neuropeptide receptors are mostly metabotropic GPCRs, and they are expressed throughout the brain and on most neurons [230, 231]. Mismatches at the scale of millimeters between neuropeptide-releasing axons and corresponding receptors are common and have defined neuropeptides as volume transmitters [19, 232]. Signaling is terminated through neuropeptide cleavage by extracellular peptidases, and by receptor desensitization and internalization [178, 179, 221, 233]. Despite important advances on release mechanisms, major knowledge gaps on neuropeptide signaling persist. The transcellular organization of release and receptors and the signaling distances are not well understood.

## Conclusions and outlook

We here assessed current knowledge on volume transmission. We focused on ascending monoamine systems and provided a comparative discussion of amino acid transmitters, acetylcholine, and neuropeptides. The central conclusion is that volume transmission mechanisms are heterogeneous, and that analyses of each system are required. Detailed, transmitter-specific knowledge on release site structure and function, on receptors, and on the relative positioning of these elements is essential for understanding neuromodulatory systems and for generating accurate models on how each modulator might control circuit function and behavior.

Major knowledge gaps persist. For dopamine, recent advances on release mechanisms now allow for generating models on signaling [23, 54]. These models need to be challenged and further developed with assessment of cell-type specific positioning of dopamine receptors. For serotonin and norepinephrine, as well as neuropeptides, more groundwork on the five domains (Fig. 2) that guide this review is necessary to build initial biophysical models of their signaling. Tool development, including of fluorescent sensors, has started delivering means for a systematic assessment of neuromodulatory transmission mechanisms [220, 234–236]. It offers new approaches to study additional modulators. For example, recent work dissected mechanisms of endocannabinoids, a family of lipophilic modulators, and surprisingly found a dependence on membrane trafficking machinery [6, 237, 238]. Ultimately, these new tools will help bridge knowledge gaps between mechanistic features of volume transmission and in vivo neuromodulatory dynamics. A molecular and cellular understanding of volume transmission will help define how these systems regulate brain function and how they can be targeted for treating disease.
